# GM-CSF Down-Regulates TLR Expression via the Transcription Factor PU.1 in Human Monocytes

**DOI:** 10.1371/journal.pone.0162667

**Published:** 2016-10-03

**Authors:** Kambis Sadeghi, Lukas Wisgrill, Isabelle Wessely, Susanne C. Diesner, Simone Schüller, Celia Dürr, Armando Heinle, Monika Sachet, Arnold Pollak, Elisabeth Förster-Waldl, Andreas Spittler

**Affiliations:** 1 Dept. of Paediatrics and Adolescent Medicine, Division of Neonatology, Paediatric Intensive Care & Neuropaediatrics, Medical University of Vienna, Vienna, Austria; 2 Dept. of Surgery, Medical University of Vienna, Vienna, Austria; 3 Core Facility Flow Cytometry, Medical University of Vienna, Vienna, Austria; University of São Paulo FMRP/USP, BRAZIL

## Abstract

Toll-like receptors (TLR) are crucial sensors of microbial agents such as bacterial or viral compounds. These receptors constitute key players in the induction of inflammation, e.g. in septic or chronic inflammatory diseases. Colony-stimulating factors (CSFs) such as granulocyte-macrophage-CSF (GM-CSF) or granulocyte-CSF (G-CSF) have been extensively investigated in their capacity to promote myelopoiesis in febrile neutropenia or to overcome immunosuppression in patients suffering from sepsis-associated neutropenia or from monocytic immunoincompetence. We report here that GM-CSF, downregulates TLR1, TLR2 and TLR4 in a time- and dose-dependent fashion in human monocytes. Diminished pathogen recognition receptor expression was accompanied by reduced downstream p38 and extracellular-signal-regulated kinase (ERK) signaling upon lipoteichoic acid (LTA) and lipopolysaccharide (LPS) binding—and accordingly led to impaired proinflammatory cytokine production. Knockdown experiments of the transcription factors PU.1 and VentX showed that GM-CSF driven effects on TLR regulation is entirely PU.1 but not VentX dependent. We further analysed monocyte TLR and CD14 expression upon exposure to the IMID® immunomodulatory drug Pomalidomide (CC-4047), a Thalidomide analogue known to downregulate PU.1. Indeed, Pomalidomide in part reversed the GM-CSF-mediated effects. Our data indicate a critical role of PU.1 in the regulation of TLR1, 2, 4 and of CD14, thus targeting PU.1 ultimately results in TLR modulation. The PU.1 mediated immunomodulatory properties of GM-CSF should be taken into consideration upon usage of GM-CSF in inflammatory or infection-related conditions.

## Introduction

Granulocyte-macrophage colony-stimulating factor (GM-CSF) is a cytokine with pleiotropic effects. It mainly acts on development and maturation of monocyte/macrophages and granulocytes. Originally this factor was found to promote myelopoiesis [1], still its overall importance for the haematological and immune system has to be discussed as mice homozygous for a disrupted GM-CSF gene develop normally and show no major perturbation of haematopoiesis. However, these mice develop lung pathology indicating that GM-CSF plays an essential role in normal lung development and—if disrupted—results in local risk to acquire infections. Consistent with latter findings, deletion of the GM-CSF receptor gene results in no relevant deficiency of myelopoiesis but in the development of pulmonary proteinosis [2]which is classified as a primary immunodeficiency [3]. Indeed, growing body of evidence suggests that GM-CSF plays an important role in infection control by maintaining emergency granulopoiesis and by improving microbicidal functions of monocytes and granulocytes [4–6]. With the aim to reduce infectious complications, CSFs have been extensively investigated in their use to promote granulopoiesis in febrile neutropenia, in myeloid reconstitution after induction and consolidation therapy of acute myeloid leukaemia (AML) or in order to accelerate reconstitution of bone marrow myeloid progenitor cells after bone marrow transplantation [7, 8]. Moreover, an influence of GM-CSF on monocytic immunocompetence during sepsis has been postulated [9].

In the clinical entity of sepsis the usage of recombinant CSFs remain controversial. Although rhG-CSF and rhGM-CSF appear to have no adverse side effects, their usefulness in treating and preventing sepsis in both adults and infants remains uncertain as CSF treatment had no impact on mortality [10]. Yet, in a monocyte HLA-DR guided pilot study immunotherapy with GM-CSF in the immunosuppressive phase of sepsis resulted in the reversal of the characteristic monocyte deactivation by restoring TLR-2 and -4 induced cytokine production [9]. While infants with severe neutropenia seem to profit from a white blood cell reconstitution, no significant reduction of mortality was observed in newborns who received GM-CSF either given prophylactically or as a treatment of an already established systemic infection [11, 12].

A growing body of evidence suggests that GM-CSF also plays an important role in the modulation of immune responses to invading pathogens [13–15]. Foreign microorganisms are detected by pathogen recognition receptors such as TLRs in the very initial phase of infection, which trigger downstream signaling cascades that converge to activate important transcription factors such as nuclear factor-κB (NF-κB) ultimately leading to production of pro-inflammatory cytokines [16].

We therefore sought to analyse the impact and mode of action of GM-CSF on innate immunity receptors sensing foreign bacteria on primary blood-derived human monocytes. We show that GM-CSF, but not G-CSF, downregulates major bacterial pattern recognition receptors including TLR1, TLR2, TLR4 and CD14 on human monocytes and that this modulation of surface receptors results in impaired downstream TLR signaling—e.g. MAPK phosphorylation. We further investigated the effects of GM-CSF on cytokine production in human monocytes upon challenge with bacterial agents such LPS or LTA.

Monocyte/macrophage differentiation is tightly regulated by transcription factors such as c-fos or PU.1 and consistently, PU.1 deficient mice show an arrest in myeloid development [17]. IMiDs® compounds (immunomodulatory drugs) pomalidomide and lenalidomide have been demonstrated to downregulate PU.1 and induce myeloid maturation arrest and neutropenia [18]. Thus, we further investigated whether pomalidomide (CC-4047) influences GM-CSF triggered TLR modulation in order to delineate the mechanisms of GM-CSF—TLR interaction and investigate possible interventions harbouring relevance for clinical use.

## Materials and Methods

### Cell isolation and culture

Heparinized whole blood was drawn from healthy adult volunteers. Human monocytes were separated by density gradient centrifugation and by means of magnetic cell sorting (Monocyte isolation kit II; Milteny Biotec, Bergisch Gladbach, Germany). Monocyte purity of negatively selected cells was > = 95% as determined by flow cytometry. Cells (1×10^6^/mL) were seeded in Teflon-coated hydrophobic culture plates (PetriPerm hydrophobic; Vivascience, Vienna, Austria) and cultured in RPMI 1640 supplemented with 10% FCS and 2 mM glutamine in the presence or absence of indicated GM-CSF concentrations.

### GM-CSF, G-CSF, pomalidomide and innate immunity ligands

GM-CSF and G-CSF was purchased from Peprotech (PeproTech GmbH, Hamburg, Germany). A stock solution of 100μg/ml was prepared in destilled water and stored in small sterile aliquots at -20°C. Effects on cell viability by high concentrations of GM-CSF were assessed by Annexin V-PE/7-AAD (Trevigen, Gaithersburg, MD) staining. The thalidomide analogue pomalidomide (CC-4047) was kindly provided by Celgene (Summit, NJ) and a stock solution of 20 mM was dissolved in DMSO. Further dilutions were made in RPMI 1640 shortly before administration to cell culture. LPS purified from *Escherichia coli* R515 was obtained from Alexis Cooperation (Lausen, Switzerland) and proven by the manufacturer to activate exclusively TLR4 as determined with splenocytes and macrophages from TLR4-deficient mice. *Staphylococcus aureus*-derived LTA (Sigma-Aldrich) was diluted in sterile distilled pyrogen-free water and stored according to manufacturer's suggestions.

### Evaluation of surface receptors by antigen staining & flow cytometry

PE-labeled anti-human TLR4 (HTA125, mouse IgG2a), TLR2 (TL2.1, mouse IgG2a), TLR1 (GD2.F4, mouse IgG1) as well as corresponding isotype antibodies were purchased from eBioscience (San Diego, CA). Anti-CD14-mAb (My4-FITC; Beckman Coulter, Fullerton, CA) was used to define the monocyte population. Antibody incubation was performed on RT for 30 min. Monocytes were washed twice with Hanks balanced salt solution (HBSS; Bio Whittaker, Verviers, Belgium) containing 0.1% NaN3, 0.3% BSA, and a total of 2×10^4^ CD14^+^ cells were analyzed by flow cytometry on a Coulter FC 500 equipped with EXPO32 software (Beckman-Coulter, Brea, CA).

### Investigation of cytokine production by intracellular cytokine staining

Intracellular tumor necrosis factor (TNF)-α cytokine staining of CD14+ cells following after 4-h incubation with LPS or LTA was performed with a PE-conjugated anti-human TNF-α mAb (mAb11, mouse IgG1). All reagents needed for detection of intracellular cytokines were purchased from BD Biosciences (San Jose, CA) and intracellular staining was performed according to manufacturer's suggestions. Intracellular TNF-α production (mean fluorescence intensity, MFI) was measured as described above by flow cytometry.

### Quantification of TLR-mediated signaling events by i.c. phospho-staining

Intracellular phospho-specific analysis of SAPK was undertaken as described previously [19]. Briefly, positively selected monocytes were either left untreated or stimulated with LPS (100 ng/mL) or LTA (10 μg/mL) for 10 min at 37°C. Fixation of phospho-epitopes was achieved using BD Phosflow Fix Buffer for 10 min at 37°C, followed by permeabilization with ice-cold 90% methanol for 30 min on ice. Cells were washed twice with staining buffer and the following phospho-specific mAb were added for 30 min at room temperature: Alexa 647-phospho-p38 (pT180/pY182, mouse IgG1, clone 36), PE-phospho-ERK1/2 (pT202/pY204, mouse IgG1), PE-phpspho-NFκB p65 (pS529, mouse IgG2b, clone K10-895.12.50) were obtained from BD Biosciences. Monocytes were stained with CD14-FITC (mouse IgG2a, clone UCH-M1; Santa Cruz Biotechnology, Santa Cruz, CA).

### RNA interference

Human primary monocytes were transfected using the Human Monocyte Nucleofector Kit (Lonza) according to the manufacturer’s instructions. Briefly, 5 × 10^6^ monocytes were resuspended into 100 μl Nucleofector solution with 0.5 nmol of either PU.1 siRNA (Darmacon, Lafayette, CO) or non-effective GFP siRNA (forward: 5′-UGACCACCCUGACCUACGGCGUGCAGUGC-3′; 5′-reverse: GCACUGCACGCCGUAGGUCAGGGUGGUCA-3′) before electroporation with the Nucleofector II Device (Lonza). Cells were then immediately removed from the device and incubated overnight with 1 ml prewarmed Human Monocyte Nucleofector Medium containing 2 mM glutamine and 10% FBS. Cells were then resuspended in complete RPMI medium and treated with appropriate cytokines to induce differentiation into macrophages.

### Quantitative TaqMan Real Time PCR

The ABI PRISM 7500HT Sequence Detection System (Applied Biosystems, NY) was used for qt-RT-PCR analysis. Primer-probes sets for TLR1, TLR2, TLR4 and PU.1 (all FAM^TM^) and 18S rRNA VIC® were obtained predesigned from Applied Biosystems and tested for primer efficacy (gene expression assays: Hs99999901_s1 18S VIC®, Hs00413978_m1 TLR1, Hs01872448_s1 TLR2, Hs00152939_m1 TLR4, Hs00162150_m1 PU.1). Multiplex amplification was carried out in a total volume of 20 μl for 40 cycles of 3 seconds at 95°C, 30 seconds at 60°C. Initial denaturation was performed for 3 min at 95°C. Target gene expression was normalized to 18s rRNA house keeping gene expression. Normalized target gene expression was analysed by the comparative ΔΔ-CT method and calculated as x-fold expression.

### Statistical analysis

Data are presented as mean ± standard deviation. Statistical analysis was performed with IBM SPSS 21. Null hypothesis was tested with one-way ANOVA adjusted according to Tukey-Kramer. *P* values are two-sided and p<0.05 was considered statistically significant. The study was approved by the Ethics Commission of the Medical University Vienna.

## Results

### TLR1, TLR2, TLR4 and CD14 expression profiles on GM-CSF treated human monocytes

**Fig 1:**
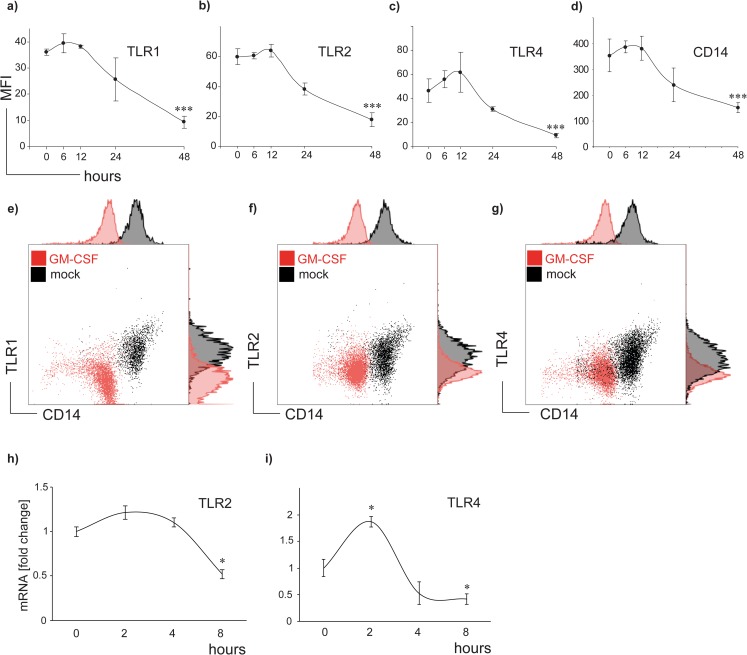
GM-CSF downregulates TLR1, TLR2, TLR4 and CD 14 in a time-dependent fashion. (a-d) Monocytes were cultured in presence or absence of 100 U/ml GM-CSF in a time course from 6,12, 24, to 48 h. TLR and CD14 mean fluorescence intensities (MFI) on human monocytes were determined by flow cytometry. Changes of mean fluorescence intensities (MFI) over time were calculated at each time point (MFIGM-CSF−MFI_medium_) and compared to baseline expression at the time point 0. Data in Fig 1 a-d represent means ± SD; *** p<0.001. (e-g) Representative flow cytometry dot plots and overlay histograms of GM-CSF treated (red) und untreated (black) CD14^+^ cells are shown for TLR1 (e), TLR2 (f) and TLR 4 (g). (h and i) mRNA transcription of TLR2 and TLR4 genes in monocytes was quantified by multiplex real-time PCR in presence or absence of 100 U/ml GM-CSF in a time course of 2, 4 and 8 hours. TLR mRNA levels were normalized to 18S house keeping gene expression and changes over time were compared to mRNA baseline expression at time point 0 which was set to 1. Data of 3 individually performed experiments show fold change ± SD; * p<0.05.

To investigate the influence of GM-CSF on TLR1, TLR2, TLR4 and CD14 protein expression, human monocytes were exposed to 100 U/ml GM-CSF in a time course of 12, 24, 48 and 72 h. GM-CSF markedly decreased TLR1, TLR2 and TLR4 expression which was accompanied by reduced CD14 levels (Fig 1A–1D). A significant effect was observed initially at 12 h and reached a maximum after 48 h (p<0.01). TLR baseline expression of untreated cells remained stable during the 48 h time period. Representative flow cytometry dot-plots and histograms are presented in Fig 1E–1G.

We further conducted real-time PCR to analyse TLR2 and TLR4 mRNA levels in human monocytes after GM-CSF exposure for 2, 4 and 8 hours (Fig 1H and 1I). A significant reduction of both gene transcripts was observed after 8 hours. Notably, TLR4 mRNA (and to a lesser extend TLR2 mRNA) expression was initially increased at the time point of 2 hours, followed by an up to 2-fold decrease after 8 hours of GM-CSF administration.

**Fig 2:**
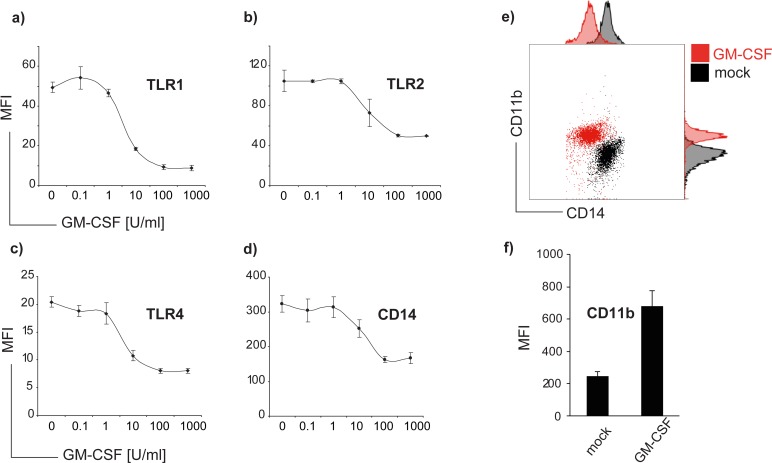
TLR and CD14 downregulation by GM-CSF is dose-dependent. Monocytes were treated with increasing doses of GM-CSF from 0.1 to 1000 U/ml for 48 hours and TLR1 (a), TLR2 (b), TLR4 (c) and CD14 (d) mean fluorescence intensities (MFI) were analysed by flow cytometry. Data of four individual experiments were analysed, error bars show means of MFI ± SD.

We next treated cells with increasing concentrations of GM-CSF ranging from 0.1–1000 U/ml for 48 h. Our results show that the effect of GM-CSF on TLR and CD14 expression is concentration-dependent (Fig 2). Down-regulation of TLR1, TLR2 and TLR4 was barely detectable at a concentration of 1 U/ml, and reached the strongest levels at a dose of 100 U/ml In contrast, CD11b was inversely regulated with highest fluorescence intensities at a concentration of 100 U/ml (Fig 2E and 2F). Administration of G-CSF neither changed TLR nor CD 14 expression (S1 Fig).

### TLR-induced p38, ERK 1/2 and p65 (RelA) phosphorylation is reduced in GM-CSF-treated monocytes

**Fig 3:**
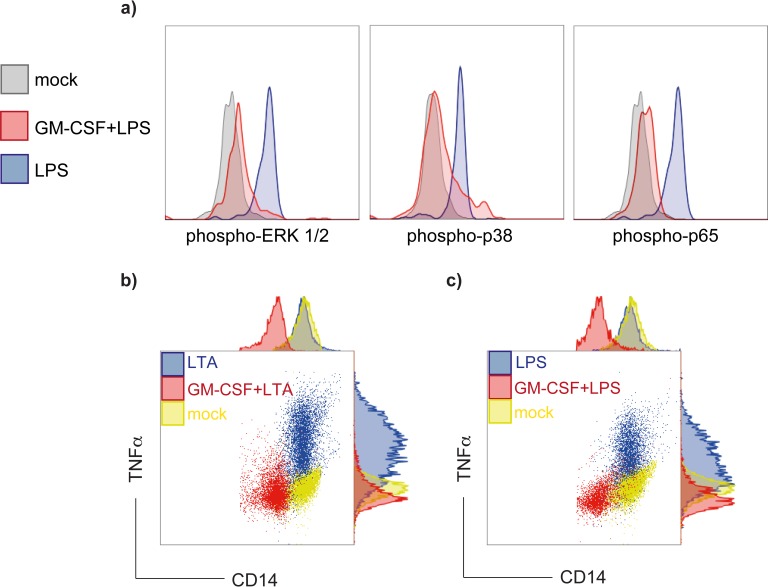
Decreased of downstream signalling protein phosphorylation and intracellular TNF-α production in GM-CSF treated CD14^+^ cells upon TLR ligand induction. (a) Negatively depleted CD14^+^ cells were stimulated for 10 min with 100 ng LPS. Cells were fixed, permeabilized and stained with phospho-specific mAb to determine p-p38, p-ERK 1/2 and p-p65 activation. Dark-gray histograms show cells treated with medium only while shaded histograms show cells challenged with LPS. GM-CSF treatment prior to TLR engagement (100U/ml, red histograms) resulted in markedly reduced phosphorylation of p38, p65 and ERK1/2. Histograms shown are representative of three individually performed experiments. (b and c) Monocytes were incubated for 48 h in absence or presence of 100 U/ml GM-CSF and afterwards stimulated for 4 h with 10 μg/mL LTA (b) or 100 ng/mL LPS (c), respectively. Cells were double-stained with CD14-FITC, TNF-α-PE or with corresponding isotype antibodies and analyzed by flow cytometry. Dot plots presented are representative from three individually performed experiments.

Engagement of TLR transmembrane proteins results in activation of a divergent network of downstream signaling events including phosphorylation of p38, ERK1/2 and the NFκB subunit p65. Using phospho-specific antibodies, we analyzed the signaling network in purified monocytes stimulated with ligands for TLR2 and TLR4, respectively. Monocytes pretreated with GM-CSF and stimulated with either LPS or LTA showed strikingly lower levels of phosporylated p38, ERK1/2 and p65 than GM-CSF untreated cells (Fig 3A), suggesting that TLR down-regulation by GM-CSF consequently impacts downstream SAPK phosphorylation.

### GM-CSF-treated monocytes show diminished proinflammatory cytokine production

Since TLR involvement ultimately leads to production of inflammatory cytokines we sought to investigate the effect of bacterial cell wall components on GM-CSF-treated monocytes. Monocytes were pretreated for 48 h with increasing concentrations of GM-CSF (0.1–1000 U/ml) and afterwards incubated with either 10 μg LTA (Fig 3B) or 10 ng LPS for 4 h (Fig 3C). TNF-α, as the surrogate cytokine of efficient TLR4 trigger, was determined by intracellular cytokine staining of CD14^+^ cells on single cell level. In GM-CSF pretreated monocytes TNF- α levels were strikingly reduced upon TLR engagement with either LPS or LTA. GM-CSF administration had no impact on cell viability (S2 Fig).

### Gene knockdown of PU.1 but not VentX abrogates GM-CSF induced TLR and CD14 downregulation

**Fig 4:**
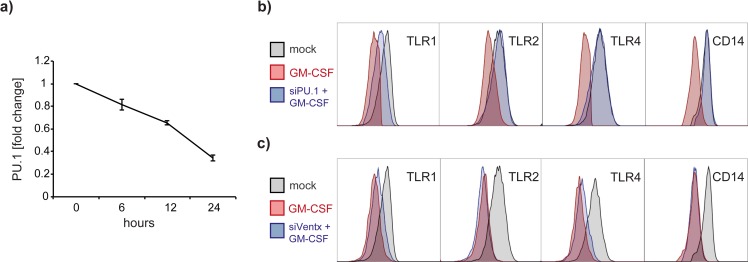
PU.1 knock down reverses CD14 and TLR downregulation in human monocytes. (a) Silencing of PU.1 results in almost 70% reduction of PU.1 mRNA levels after a time period of 24 hours. (b) Overlay histograms of GM-CSF treated human monocytes GM-CSF-induced TLR downregulation (dark-grey histograms) is abrogated in PU.1 silenced cells (blue histograms). Shaded histograms represent TLR baseline expression in untreated cells. (c) Silencing of VentX, however, did not reverse GM-CSF induced TLR-downregulation in human monocytes. Overlay histograms shown are representative for three individually performed experiments.

Multiple transcription factors have been implicated in monocyte activation and therefore might also govern TLR expression. To elaborate the mechanisms underlying GM-CSF-mediated TLR regulation, we employed siRNA technology to knock down the transcription factors PU.1 and VentX expression in primary monocytes. As shown in Fig 4A silencing PU.1 resulted in 3-fold downregulation of PU.1 RNA levels within a 24 hours time frame. Remarkably, PU.1 silenced cells were unresponsive to GM-CSF in terms of TLR1, TLR2, TLR4 and CD14 expression patterns (Fig 4B). PU.1 silencing alone did not change TLR expression levels (data not shown). In contrast, knock down of the homeobox transcription factor VentX, did not revert the GM-CSF-induced TLR downregulation (Fig 4C).

### Pomalidomide partly reverses TLR and CD14 downregulation

**Fig 5:**
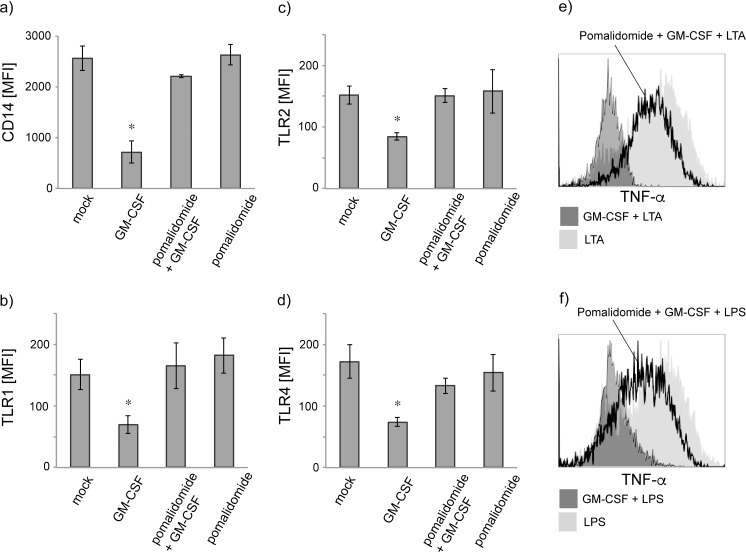
Pomalidomide counters GM-CSF-triggered CD14 and TLR downregulation and partly reverts the GM-CSF-induced effects on cytokine production. (a-d) Treatment with pomalidomide alone did not change monocyte TLR and CD 14 but revert the GM-CSF-induced downregulation of TLR1 (a), TLR2 (b), TLR4 (c) and CD14 (d). (e and f) Cytokine staining on single cell level by flow cytometry shows that presence of GM-CSF (dark grey histograms) inhibited TNF-α cytokine production in CD14^+^ monocytes upon LPS (e) or LTA (f) stimulation. Additional administration of pomalidomide prior to GM-CSF partly reverted the GM-CSF-induced dampening of proinflammatory cytokine production after LPS or LTA challenge (open black histograms). Shaded histograms show robust TNF-α production in monocytes stimulated with LTA or LPS alone. Histograms shown are from three individually performed experiments.

We next questioned whether pharmacological agents that regulate the transcription factor PU.1 also affect GM-CSF-mediated TLR downregulation. The IMiD Pomalidomide has been shown to down-regulate PU.1 in leukocytes both in vitro and in vivo [18] and to arrest myeloid maturation. We therefore pretreated primary monocytes with pomalidomide (10 μM) for 4 hours prior to GM-CSF administration for 48 hours. As shown in Fig 5 addition of pomalidomide before GM-CSF treatment significantly inhibited the GM-CSF effects and in part reverted TLR1 (a), TLR2 (b), TLR4 (c) and CD14 (d) downregulation. Accordingly, no differences in intracellular TNF-α production were observed in cells cultured with both pomalidomide and GM-CSF (e and f). Pomalidomide alone did neither change TLR nor CD14 expression patterns.

## Discussion

Toll-like receptors are of major importance in their function as PRRs and thus fulfil the recognition of microbial agents with concomitant immunological stimulation. They have been widely shown to induce an inflammatory response upon pathogen-associated molecular patters (PAMP)- and damage-associated molecular patters (DAMP)-contact and they bridge signalling events between the innate and the adaptive immune response [20, 21]. However, intrinsic and extrinsic regulation of TLRs is far from being understood in detail.

Since the discovery of the human Toll homologues in the mid 1990s a considerable number of TLR-modifying agents have been described. We previously showed that 1,25-cholecalcitriol—via the Vitamin-D-receptor pathway—is a potent down-regulator of TLR2 and TLR4 in mononuclear cells [22]; other agents that modulate TLR downstream signalling include a number of microRNAs (e.g. miR-146a) [23] or all-trans retinoic acid [24]. The three colony-stimulating factors, granulocyte/macrophage colony-stimulating factor (GM-CSF), macrophage colony-stimulating factor (M-CSF), and granulocyte colony-stimulating factor (G-CSF), have been regarded as immunostimulators because of their role in myeloid hematopoiesis and immune function. Here we show that GM-CSF also possesses distinct immunosuppressive effects on human monocytes by hampering cytokine production through the TLR axis. Regarding the pleiotropic effects of GM-CSF in humans an essential impact of GM-CSF applications *in-vivo* on processes from hematopoesis, to angiogenesis, to cellular functionality harbour relevant implications [25]. Recent reports argue that the function of colony stimulating factors, including GM-CSF, should be differentiated in autocrine and paracrine functions. Moreover, a functional discrimination appears to be necessary depending on the tissue of action [15, 26]. All these caveats have to be taken into account when considering GM-CSF as immunomodulator in a clinical setting. GM-CSF unfolds its biological effects through a number of transcription factors, among these are c-fos, PU.1 and VentX [27]. In pulmonary macrophages both phagocytosis and response to LPS is regulated by GM-CSF via PU.1 [14, 15] and indeed, targeting PU.1 preserved GM-CSF mediated TLR downregulation. Remarkably, analysis of 5'-proximal promotor region of the human TLR4 gene revealed a PU.1 binding site, indicating that PU.1 participates in the basal regulation of TLR4 [28]. Alongside, the homeobox transcription factor VentX has recently been shown to strongly impact the proinflammatory properties of macrophages and to impair their differentiation. However, in our experiments we didn’t find any evidence that VentX is additionally involved in GM-CSF-promoted TLR regulation. VentX silencing did not influence TLR expression indicating that this transcription factor is involved in the M-CSF and G-CSF rather than the GM-CSF signaling route.

The ever expanding knowledge of the role of TLRs in the pathogenesis of various human diseases make TLRs promising therapeutic targets. The contribution of TLRs in the pathogenesis of atherosclerosis or inflammatory bowel diseases is well documented. [29, 30] In the initial phase of sepsis, where an overwhelming number of pathogens leads to a severe systemic inflammatory response, it might prove beneficial to hamper the inappropriately overactive inflammation. On the other hand, in patients suffering from sepsis who have entered the immnosuppressive state of the disorder—as identified by reduced HLA-DR expression and by diminished ex vivo LPS-driven TNF-α production—administration of GM-CSF led to a restoration of the monocyte function. In these patients, an increase of monocyte HLA-DR expression and a restored stimulatory capacity measured by TNF-α production upon LPS contact could be detected [9]. Therefore, GM-CSF might prove its worthiness in biomarker-guided treatment in patients with sepsis by its immunomodulatory properties and by restoring white blood cell numbers in neutropenia patients.

Pomalidomide (CC-4047) is a thalidomide analogue with immunomodulatory properties. IMiD® compounds pomalidomide and lenalidomide are currently experiencing a renaissance as anti cancer agents for treatment of myelodysplastic syndromes (MDS) and for treatment of patients with multiple myeloma in combination with dexamethasone. These drugs exhibit a range of interesting clinical properties, including anti-angiogenic and anti-proliferative activities although exact cellular targets are only partly understood. Lenalidomide and pomalidomide strongly inhibit T-regulatory cell proliferation and suppressor-function [31]. Also, anti-inflammatory effects on LPS-stimulated monocytes such as decreased TNF-α production have already been described. In our experiments pomalidomide counter regulated GM-CSF modulated responsiveness to LPS and LTA in primary human monocytes. Pomalidomide has been shown to downregulate PU.1 in both human osteoclasts and human granulocytes [18, 32]. Most likely, the diminished response to PAMPs such as LPS or LTA is mediated by TLR2 and TLR4 downregulation, which is PU.1 transcription factor-driven.

In summary, our findings indicate a critical role of PU.1 in the regulation of TLR1, 2, 4 and of CD14, thus targeting PU.1 ultimately results in TLR modulation. Thus, we identified a novel immunomodulatory function of GM-CSF in human blood derived monocytes. The dampening of proinflammatory TLR signaling pathways might be beneficial in diseases where excessive inflammation leads to harmful outcome. These mechanisms of GM-CSF and TLR interaction might be of relevance for the clinical use of a pleiotropic colony stimulating factor.

## Supporting Information

S1 Fig G-CSF does neither alter CD14 nor TLR surface expression on human monocytes. Monocytes were isolated as described in methods and incubated for 48h in the presence or absence of recombinant G-CSF. Cells were harvested and analyzed for CD14, TLR1, TLR2 and TLR4 surface protein by flow cytometry. Error bars show mean fluorescence intensities (MFI) ± SD of three individual experiments.
(TIF)

S2 Fig GM-CSF does not change cell viability. Monocytes were incubated with or without GM-CSF for 48 hours and Annexin V / 7 AAD staining was performed. Flow cytometry dot plots shown are representative of three individually performed experiments.
(TIF)

## Abbreviations

AML: acute myeloid leukaemia
DMSO: Dimethyl sulfoxide
ERK: extracellular-signal-regulated kinase
G-CSF: granulocyte-colony stimulating factor
GFP: green fluorescence protein
GM-CSF: granulocyte-macrophage-colony stimulating factor
HBSS: Hanks balanced salt solution
IMID: immunomodulatory drug
LPS: lipopolysaccharide
LTA: lipoteichoic acid
MAPK: mitogen-activated protein kinase
NF-κB: nuclear factor-κB
PAMP: pathogen-associated molecular pattern
PI: propidium iodide
SAPK: stress-activated protein kinase
TNF: tumor necrosis factor
TLR: Toll-like receptor
