# Supplementary figures and images for: GM-CSF Down-Regulates TLR Expression via the Transcription Factor PU.1 in Human Monocytes
Source: PLoS One. 2016 Oct 3;11(10):e0162667. doi: 10.1371/journal.pone.0162667 (PMC5047522; doi:10.1371/journal.pone.0162667)

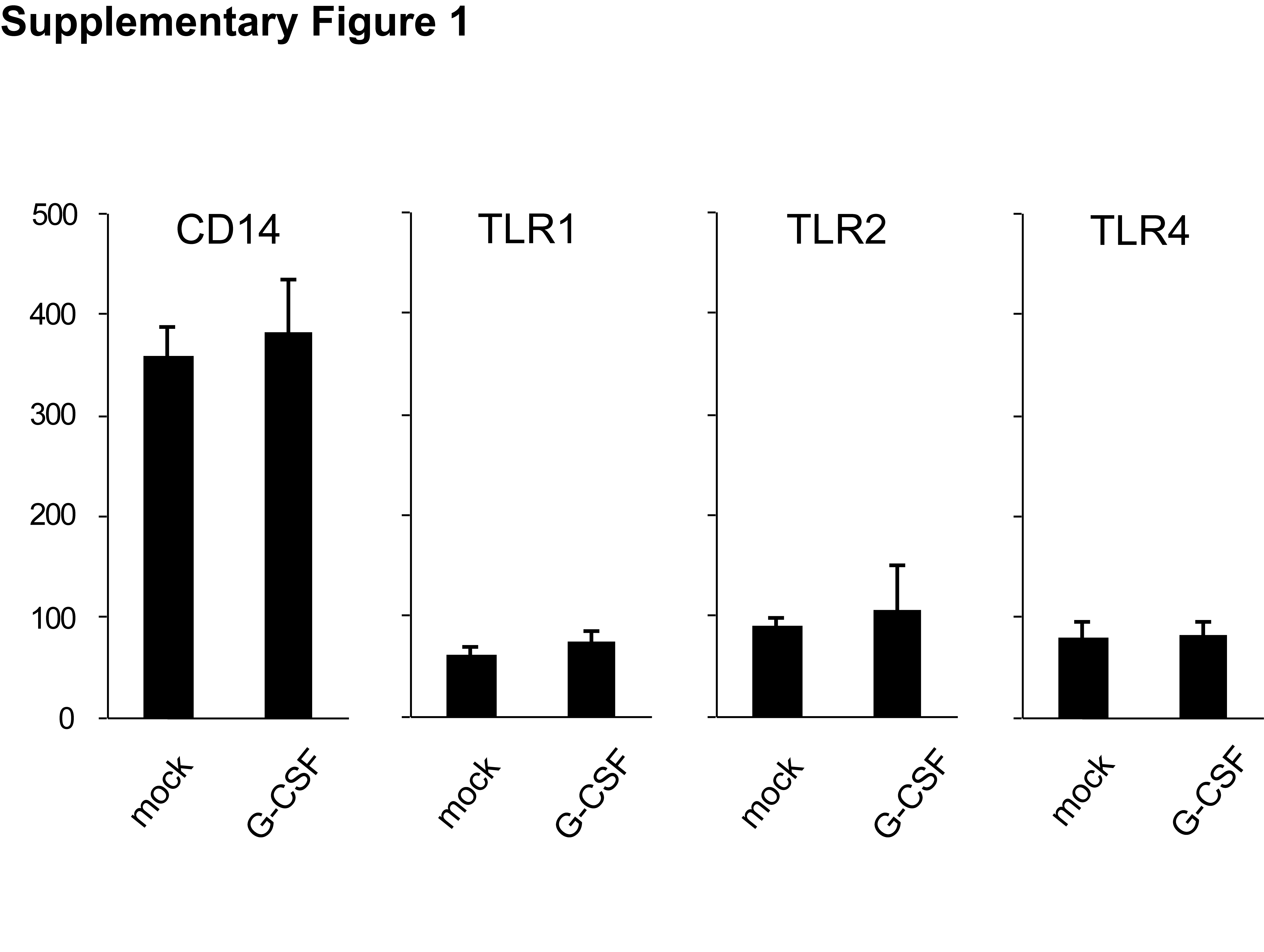

Supplement: S1 Fig — Monocytes were isolated as described in methods and incubated for 48h in the presence or absence of recombinant G-CSF. Cells were harvested and analyzed for CD14, TLR1, TLR2 and TLR4 surface protein by flow cytometry. Error bars show mean fluorescence intensities (MFI) ± SD of three individual experiments. (TIF) [file pone.0162667.s001.tif]

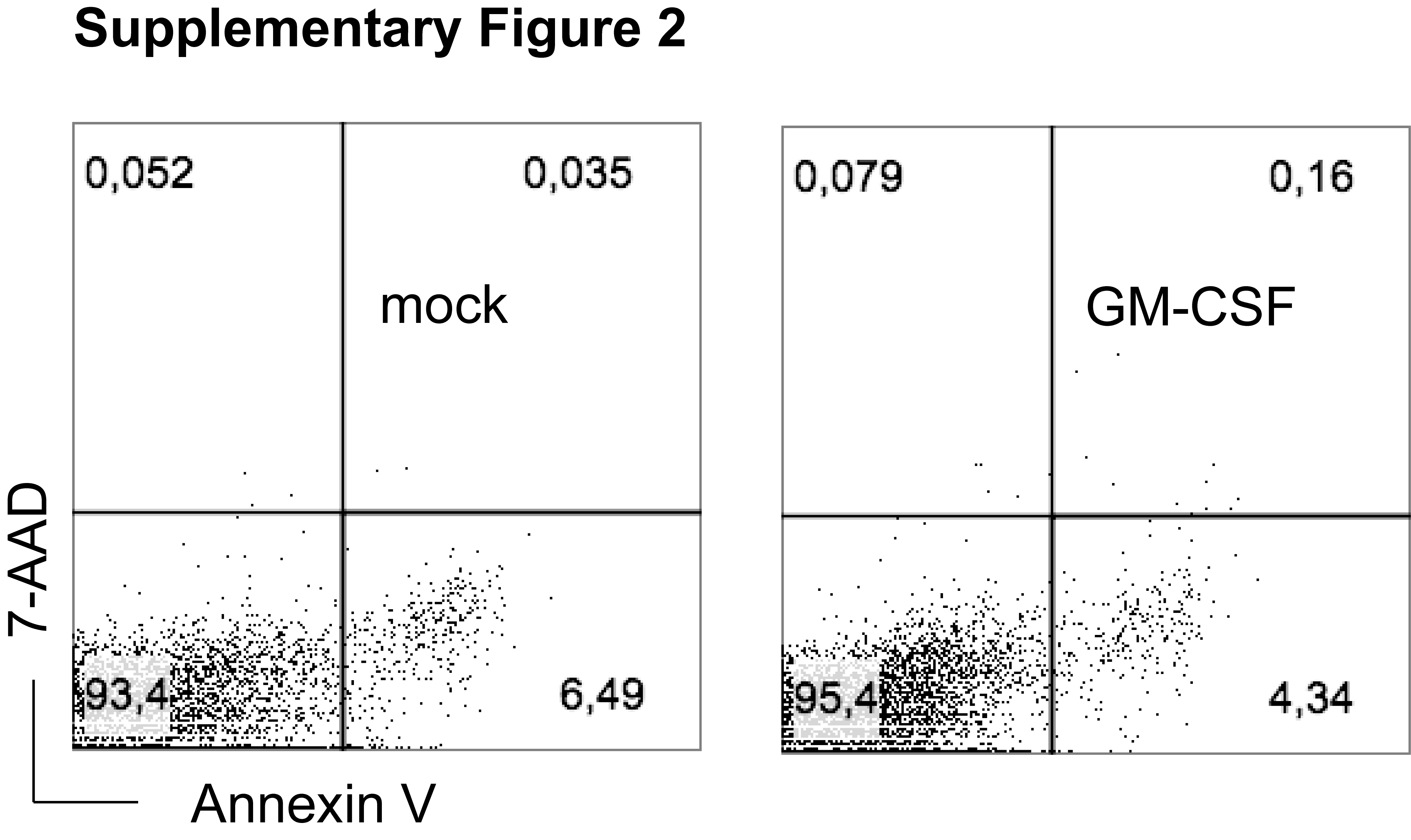

Supplement: S2 Fig — Monocytes were incubated with or without GM-CSF for 48 hours and Annexin V / 7 AAD staining was performed. Flow cytometry dot plots shown are representative of three individually performed experiments. (TIF) [file pone.0162667.s002.tif]
